# A simple on-field fast knee-flexion test to assess acute knee flexor fatigue

**DOI:** 10.1007/s00421-025-05732-2

**Published:** 2025-04-19

**Authors:** Cornelis J. de Ruiter, Lucas M. Baak, Yfke Westerling, Erik Wilmes

**Affiliations:** 1 Department of Human Movement Sciences, Faculty of Behavioural and Movement Sciences, Vrije Universiteit Amsterdam, Van der Boechorststraat 9, 1081 BT Amsterdam, The Netherlands; 2 FIFA Medical Centre of Excellence, Royal Netherlands Football Association, Woudenbergseweg 56-58, 3707 HX Zeist, The Netherlands

**Keywords:** Rapid force development, Football, Sprinting, Muscle fatigue, Butt-kicks

## Abstract

**Purpose:**

In a practical setting, outside the laboratory, acute muscle fatigue may be underestimated because substantial recovery occurs during the elapsed time between the end of exercise and fatigue assessment. We introduce a simple field test to assess knee flexor contractile function quickly after exercise cessation.

**Methods:**

Fourteen young amateur football players performed maximally fast knee flexions (FKFs) in the prone position with their dominant leg, before (pre) and 20 s after finishing a series of fourteen fatiguing 40 m sprints (post) and again following 6 min recovery (rec). Peak angular acceleration (PAA) about the knee joint was measured with a small inertial measurement unit (IMU) firmly attached to the shin.

**Results:**

Although participants only practiced the FKFs for 1 min in the warm-up, the reliability of PPA was good with coefficients of variation of 3.0% (pre), 2.7% (post), and 3.6% (rec). Sprint time increased from 5.96 ± 0.40 s to 6.55 ± 0.37 s (*p* < 0.001, *f* = 0.89), PAA decreased from 107.1 ± 11.5 rad.s^−2^ to 94.1 ± 11.7 rad.s^−2^ (*p* < 0.001, *f* = 0.50) and following recovery (*p* < 0.05) values were 6.15 ± 0.39 s and 103.1 ± 10.7 rad.s^−2^, respectively. The percentage decrease in PAA during FKFs was linearly related (*r*^2^ = 0.48, *p* = 0.01) to the percentage increase in 40 m sprint time. In addition, PAA (pre) was related to the time of the first sprint (*r*^2^ = 0.33, *p* = 0.03).

**Conclusion:**

The proposed FKF test is reliable and can easily be executed to evaluate acute knee flexor muscle fatigue on the field. The presented relations between (changes in) sprint performance and peak knee angular accelerations during isolated fast knee flexions are promising but need confirmation in larger-scaled studies.

**Supplementary Information:**

The online version contains supplementary material available at 10.1007/s00421-025-05732-2.

## Introduction

Hamstring injuries present a great problem in sports, hampering athletes’ careers and posing clubs with the financial costs of rehabilitation and missed matches, its prevalence in football has increased during recent seasons (Ekstrand et al. 2022). Hamstring injuries predominantly occur during sprinting and their incidence has been related to fatigue (Woods et al. 2004; Ekstrand et al. 2011; Della Villa et al. 2023). This has motivated researchers to study neuromuscular knee flexor fatigue outside the laboratory in a practical setting (Marshall et al. 2014; Wilmes et al. 2021). The study of fatigue is also interesting because of its relationship with performance decline. Fatigue has been defined as ‘any reduction in the maximal capacity to generate force or power output’ (Vollestad 1997), operationalized in the present study for the knee flexors as a reduction of ‘unloaded’ angular knee flexion acceleration and for sprint runs as an increase in sprint time. In football, knee flexor muscle contractile function deteriorates at half-time and post-matches during 11 versus 11 players and simulated matches (Wilmes et al. 2021; Marshall et al. 2014; Rahnama et al. 2003).

A major limitation of assessing neuromuscular fatigue in applied settings is the time it takes to place players in equipment used to measure contractile muscle function. Probably, acute fatigue is largely caused by metabolic changes in the muscle (Sundberg and Fitts 2019) and is known to show quick recovery (de Ruiter et al. 2000; Bishop et al. 2011; Jones 2010). With simulated matches and using a special on-field dynamometer, we recently measured knee flexor force 60 s after the last activity (Wilmes et al. 2021), other studies do not always explicitly report this time (Rahnama et al. 2003; Marshall et al. 2014), but 60 s will approximately be the lower limit. This may lead to underestimated fatigue, especially following intensive play, because recovery can be substantial in 60 s (de Ruiter et al. 2000; Bishop et al. 2011; Jones 2010). Thus, although decreased post-match knee flexor muscle strength has been reported (Rahnama et al. 2003; Marshall et al. 2014; Wilmes et al. 2021), it seems plausible that fatigue was underestimated in these studies. Since hamstring injuries are highly prevalent during running (Woods et al. 2004) and maximal sprinting (Askling et al. 2007), the risk of sustaining an acute hamstring injury might be greater during periods of intense play especially in the fatigued state (Woods et al. 2004; Ekstrand et al. 2011; Della Villa et al. 2023). Therefore, it would be valuable to assess fatigue, immediately following intense periods of play, when the metabolic milieu and muscle contractile capacity may differ substantially from those measured one to several minutes later (Bogdanis et al. 1995; Karatzaferi et al. 2001).

In the present study, we propose using on-field fast knee flexions (FKFs) executed in a prone position to assess knee flexor contractile function rapidly following intense exercise. The knee flexion test only requires players to wear a small inertial measurement unit (IMU) on the shank without further equipment, while a second IMU on a timing gate will be used to assess sprint time in the present study.

We reasoned that peak angular acceleration (PAA) during maximally fast ‘unloaded’ knee flexion, represents the knee extensors’ capacity for rapid force development necessary to get the mass of the lower leg into motion. Others have studied maximally fast unloaded contractions during seated knee extensions in the laboratory. These were also executed without additional external resistance and ended in a cushion to decelerate the lower limb (Houston et al. 1988; Andersen et al. 2005). Andersen et al. explained the importance of rotational acceleration of the lower leg because it affects both knee moment and knee flexion/extension velocity (Andersen et al. 2005). In maximally fast unloaded knee extension or flexion, the total knee moment is the sum of the moment due to gravitational forces and the moment due to inertial forces (i.e., the moment directly related to rotational acceleration). In addition, the rotational peak velocity is reached much earlier (after about 90 ms) in unloaded knee extension than peak muscle force (after about 300 ms). Therefore, fast force development is important for maximal rotational acceleration and knee power (i.e., the product of knee moment and rotational velocity). Houston et al. found a positive correlation (r = 0.68) between peak angular acceleration (reached in about 10 ms) and the percentage of fast twitch fibre area in the vastus lateralis muscle (Houston et al. 1988). Together these results illustrate the importance of contraction onset, which is in line with studies on the very early phase (40-100 ms) of isometric force development (Folland et al. 2014) that in addition was shown to be related to vertical jump performance (de Ruiter et al. 2007, 2006).

Since fatigue is known to reduce both the force and speed of muscle contraction, PAA is expected to decrease with fatigue, and importantly, especially with short-term fatigue, PAA should largely recover after a few minutes of rest (Bogdanis et al. 1995; de Ruiter et al. 2000; Jones 2010; Bishop et al. 2011). An important requirement of any test assessing acute muscle fatigue is a low intra-session variability. If a test is easy to perform, this will reduce within-participant variability and increase test sensitivity.

Based on pilot experiments, we hypothesize that maximally fast knee flexions in a prone position are easy to execute and standardize. Consequently, PAA expects to have a low coefficient of variation and changes in PAA can be used to assess sprint-induced knee flexor fatigue quickly following exercise. The maximal shortening velocity of isolated muscle contractions is known to decrease with fatigue ( e.g. (de Ruiter et al. 2000)) and during fatiguing repetitive sprints speed, step frequency, and knee angular velocity decrease (e.g. (de Ruiter et al. 2022)). Therefore, we also anticipated PAA in the knee flexion test to decline with fatigue. In addition, the participants will differ in sprint capacity and fatigue resistance, therefore, we also hypothesize a significant relation between the increase in sprint time and the decrease in PAA among participants.

## Methods

### Participants

Initially, seventeen amateur football players from different teams volunteered for the study (15 males, and 2 females). However, due to technical problems, the IMU data of two participants were incomplete and one participant sustained an ankle sprain during the measurements. Therefore, the final data set (one female) comprises 14 participants (age: 23.1 ± 2.3 years, body mass: 77.2 ± 6.0 kg, height: 1.85 ± 0.04 m) with an average playing experience of 14.0 ± 4.5 years. They participated after providing informed consent. Among the players were seven defenders, four midfielders, and three attackers. They were injury-free for at least three months before the measurements and did not sustain a hamstring injury in the last twelve months. They engaged in one to two training sessions and one match per week. The study was done following the Declaration of Helsinki (2013) and received approval from the local scientific and ethics review board (VCWE-2019-070R1). All participants were individually (separately) tested. The study was conducted on outdoor artificial turf and the surface was dry. Weather conditions varied with a mean temperature of 14 ^◦^C (range 8–22), a mean wind speed of 17.6 km.h^−1^ (range 12–30), and humidity of 69.1% (51–89).

### Preparation and warm-up

Procedures were once more explained on the test day. The preferred back leg of the participant when attaining a split stance position before a sprint start was defined as the dominant leg. The skin over the tibial bone of this leg was shaved for proper fixation of the shank-IMU after the warm-up and the participant put on a compression calf sleeve (Hansaplast Sport, Beiersdorf, Hamburg Germany) used to fixate the IMU. After this, a supervised warm-up (15–20 min) was conducted, which included 4 min of jogging with football-specific exercises, some basic stretching, and 4 min of strength exercises, during which the participants also practiced correct execution of maximally fast unloaded knee flexions (‘butt-kicks’). The warm-up ended with three 45 m sprints passing through the mechanical timing gates (see below) executed with increasing (60%, 80%, and 100%) voluntary effort.

### Inertial measurement units (IMUs)

Following the warm-up IMUs (MPU-9150, Invensense, San Jose, CA, USA) were switched on and time-synchronized with a mechanical thud. The IMUs continuously sampled 3D linear acceleration, 3D angular velocity, and 3D magnetic field strength at 500 Hz. The data was stored on an internal SD card. One IMU was fixated on the tibial bone of the dominant leg below the knee joint at about one-third of the distance between the knee and ankle while avoiding contact with muscles. First, a stretch of Leukotape (12 × 5 cm) was applied as a ground layer to which the IMU was firmly attached using a double-sided Nanotape, covered with Fixomull Stretch (12 × 5 cm) and covered by the compression calf sleeve. This was followed by a standard IMU-to-shank calibration procedure requiring participants to stand still and upright for 10 s to determine the longitudinal axis of the shank, followed by a thigh rise in the sagittal plane to determine the frontal axis as detailed by Wilmes et al. (Wilmes et al. 2020). The second IMU was fixated with double-sided tape to a mechanical timing gate 40 m from the start, detecting the finish moments from the peak in the IMU signals caused when the participants passed through its light 1 m long swivel as detailed elsewhere (de Ruiter and van Dieen 2019).

### Sprint and fast knee flexion protocol

Five minutes following warm-up, participants executed 14 maximal 45 m-sprints starting every 50 s (5 s countdown started at t = 45 s), with the foot of the non-dominant leg at the starting line. Following deceleration after a cone placed at 45 m (time gate at 40 m), they jogged back to the start line. Continuous verbal encouragement urged participants to go all out. Six min and 20 s after the finish of sprint number 14 one additional sprint was executed to assess sprint recovery.

The first sequence of five FKFs (details below) was done 2 min before the start of the first sprint (pre), and a second sequence was started 20 s after the finish of sprint 14 (post). A third sequence started 5 min after the finish of sprint 14 to assess knee flexion recovery (rec). After that, the participant walked back to the start line and 6 min and 20 s after the 14th sprint, executed a final 15th sprint to assess sprint recovery. Before each FKF sequence, the Rating of Perceived Exertion (RPE) was recorded on a ten-point scale (Arney et al. 2019).

### Details of fast knee flexion execution

**Fig. 1:**
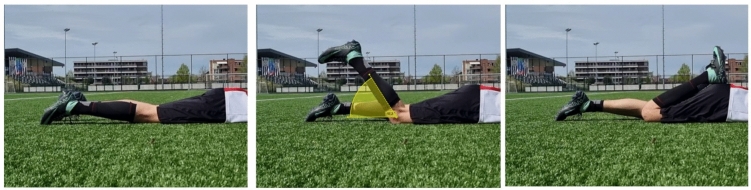
Fast Knee Flexion execution. From left to right: FKFs start position, position after ~ 1 rad knee flexion and at maximal flexion hitting the buttocks

Participants laid down 15 m after the timing gate in a prone position with straight legs, their feet in plantar flexion and their chin resting on their folded arms. From this relaxed position, they were instructed to flex the knee of the dominant leg as fast as possible: ‘kick your butt as fast as possible’ (Fig. 1). They were urged not to decelerate the lower leg: ‘Let your heel bounce back from your buttocks’. All FKFs were video recorded in the sagittal plane with a smartphone (Samsung Galaxy S21, 30 Hz) placed at 1.5 m and 0.3 m in height. This was done to corroborate correct execution: a fluent fast knee flexion during which the upper leg had to remain in contact with the field and the participant’s heel had to bounce back from the buttocks.

### Data analysis

Custom-written Matlab (version R2022b, The Mathworks, Inc., Natick, MA, USA) scripts were utilized for data analysis. The orientation of the shank IMU was continuously estimated using a gradient descent Madgwick algorithm (Madgwick et al. 2011). Together with the data of the IMU-to-shank calibration procedure, the 3D orientation of the shank was derived. The complete procedure has been described elsewhere in detail (Wilmes et al. 2020). Raw IMU data and estimated angular shank speed about the frontal axis (sagittal body plane) were stored in Matlab struct configurations and further analysed with custom-written Python (Version 3.11) scripts. These Python scripts comprised the automatized detection and extraction of FKFs and sprints.

Shank rotational speed about the frontal knee axis was 12 Hz low pass filtered using a second-order Butterworth filter. Sprint start time was defined as the first moment the shanks’ rotational speed became > 1 rad.s^−1^ during the start (the back leg always moves first when starting from a three-point position). Finish time was defined as the instance where the vector norm of the linear acceleration signals of the time-gate-IMU became > 20 m.s^−2^ (de Ruiter and van Dieen 2019).

Since during FKFs, the thigh remains horizontal, angular shank IMU speed is equivalent to angular knee flexion speed. The onset of the FKF movement was defined as the last moment before knee flexion where angular shank speed was < 0.05 rad.s^−1^. The resting shank angle was defined as the mean shank angle during the 400 to 100 ms time interval before onset. Attempts were deemed valid if the deviation from the resting shank angle during the 40 ms time interval before onset was less < pi /180 radians (= 1 degree), confirming the absence of meaningful (counter-) movement before onset. Angular knee flexion acceleration was the time derivative of angular speed.

### Statistical analysis

**Fig. 2:**
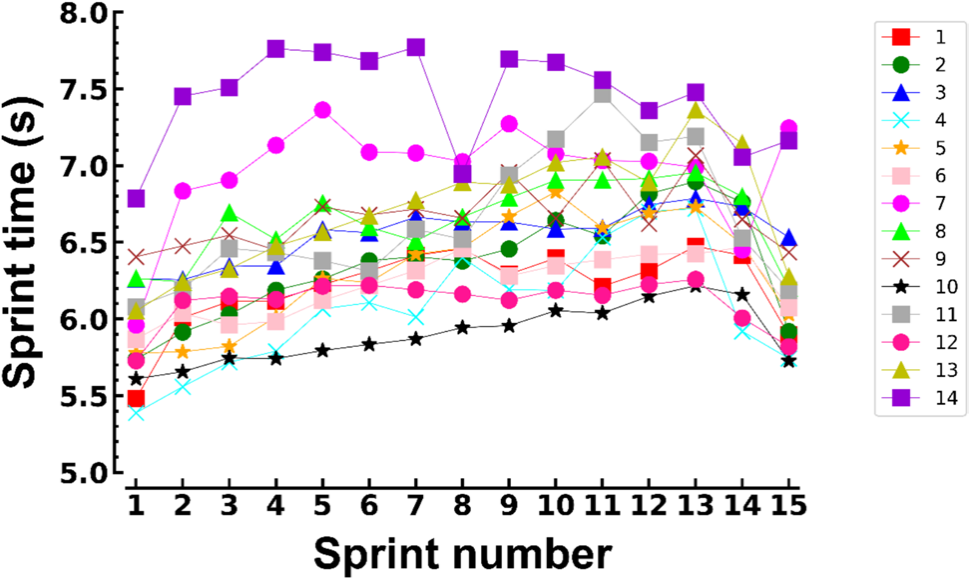
Sprint times as a function of sprint number for individual participants (1–14). Sprint number 14 was the last sprint of the fatiguing series and number 15 was executed following 6 min recovery. Note that the time of sprint 8 of participant 14 isn’t a measurement error, and participant number 7 did not sprint maximally following the 6-min recovery period

Because the FKF test is new, a priori power calculation (α = 0.05, β = 0.95) was based on the expected sprint time effect using GPower 3.1 (Faul et al. 2007). We made conservative choices by including only the most interesting sprints (numbers 1, 13, 14, and 15) in this calculation and using a moderate effect size estimation (*f* = 0.5) compared to other studies (> 1.0) (van den Tillaar 2018; Jimenez-Reyes et al. 2019). The estimated number of participants was 10 but given the uncertainties with the repeatability of the FKF test and possible dropout, we included 17 participants. As indicated in the above we report the data of 14 participants. However, for the recovery sprint (number 15), for reasons unknown participant number 7 did not sprint maximally (Fig. 2) this data point was an outlier and excluded from the analysis.

The mean values of the three attempts (out of five) with the greatest PAA were used to quantify changes over time (pre, post, and after recovery).

Normal distribution of the data was checked using Q-Q plots, histograms, and the Shapiro–Wilk test. To assess the statistical significance of fatigue and recovery, sprint numbers 1 (pre), 14 (post), and 15 (recovery) were included in separate repeated measures ANOVA for sprint times and PAA. For sprint time the pen-ultimate sprint (13) results were added in this analysis to investigate the presence of an end-spurt (Foster et al. 2023). On significance (*p* < 0.05) this was followed by Bonferroni corrected post hoc testing. Effect sizes are reported as Cohen’s f and d values.

RPE was evaluated with the non-parametric Friedman test (Kendall’s W is reported as rank correlation) followed by a Bonferroni-adjusted Wilcoxon test.

To obtain the reliability of the FKF test, for each of the three conditions separately (pre- and post-sprinting and following recovery), a two-way random Intra Class Correlation coefficient (ICC(2,1), agreement) and a coefficient of variation (CV) were calculated, using the three attempts with the greatest PAAs in each condition. CVs have been classified as highly reliable (≤ 5.0%), moderately reliable (5.1–10.0%), or unacceptable (> 10.0%). ICC values have qualitatively been described as poor (< 0.50), moderate (0.50–0.74), good (0.75–0.90), or excellent (> 0.90) (Koo and Li 2016).

Pearson’s correlation coefficient was calculated to establish the significance of correlation and reported as coefficients of determination (r^2^).

## Results

### RPE

The Rating of Perceived Exertion (RPE) increased (W = 0.96, *p* < 0.001) from a median value of 3 (range 2–5) before the sprint series to 9 (range 8–10) immediately after the fourteenth sprint (*p* = 0.003). RPE was not completely recovered following 6 min of rest (median 4, range 2–6, *p* = 0.01).

### Sprint times

Most participants showed the expected gradual increase in sprint time from sprint 1 to 14. However, this pattern varied among participants (Fig. 2). ANOVA repeated measures (without participant 7 who did not sprint all-out after recovery) for the sprints of interest (1, 13, 14, and 15), showed a significant main effect of sprint number on 40 m times (F_3,36_ = 56.7, *p* < 0.001, *f* = 0.89). Mean (± standard deviations, n = 13) sprint times were 5.96 ± 0.40 s, 6.81 ± 0.40 s, 6.55 ± 0.37 s and 6.15 ± 0.39 s, respectively. All pairwise post hoc comparisons were significant, with effect sizes ranging from 1.06 to 2.85. Consistent with the end-spurt phenomenon, sprint 14 was faster than sprint number 13 (*p* = 0.002, d = 1.06). There was recovery as sprint 15 was faster than sprint 14 (*p* = 0.001, d = 1.43), but recovery was incomplete since sprint 15 still was slower than sprint 1 (*p* = 0.002, d = 1.41).

### Peak angular acceleration during fast knee flexions

**Fig. 3:**
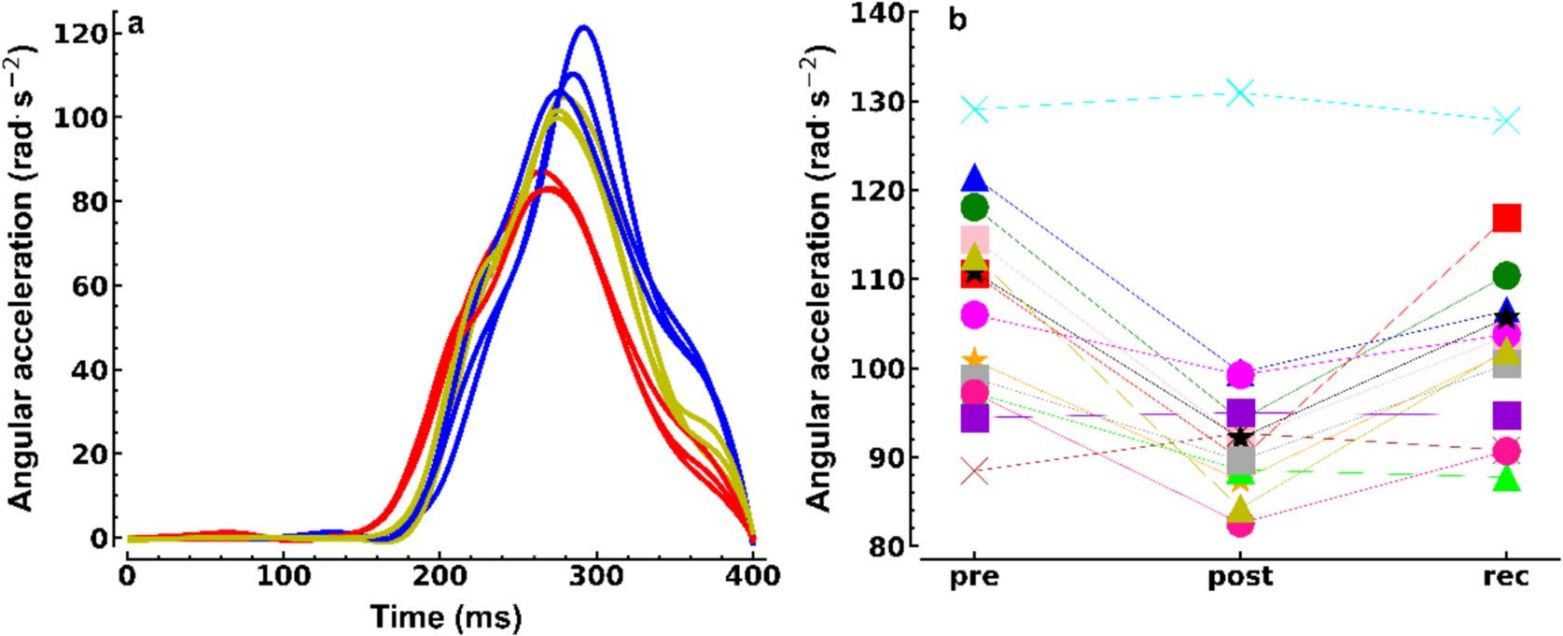
**a** Example of knee angular acceleration as a function of time (participant 13). The three fastest Knee Flexion attempts pre- (blue), post-sprinting (red), and following recovery (olive) are depicted. Traces are aligned at *t* = 400 ms (zero crossings of the acceleration signals). **b** Individual (*n* = 14, for labels, see Fig. 2) peak knee angular accelerations pre-, post-sprinting, and following recovery (rec). Each data point is the mean value of the three attempts. Standard deviations are omitted for clarity

Figure 3a shows example data of the angular acceleration signals during the FKFs. ICCs (95% confidence interval) pre-sprints, post-sprints, and following recovery were 0.82 (0.44–0.93), 0.86 (0.48–0.95), and 0.79 (0.48–0.91), respectively. The CVs in the three conditions were 3.0%, 2.7%, and 3.6%.

PAA (rad.s^−2^) significantly decreased (F_2,26_ = 16.2, *p* < 0.001, *f* = 0.50) from 107.1 ± 11.5 (pre) to 94.1 ± 11.7 post-sprinting (*p* = 0.001, d = 1.26) and it recovered to 103.1 ± 10.7 (*p* = 0.006, d = 1.0). Although recovery values were not significantly different from the pre-sprint values (*p* = 0.08, d = 0.66), recovery appeared incomplete, but less so than for the sprint times. Similar to changes in sprint time, the sprint-induced changes in PAA during the FKFs also varied considerably among participants (Fig. 3b).

The knee angle at which PAA (rad) was obtained significantly increased (F_2,26_ = 8.0, *p* = 0.002, *f* = 0.15) from 0.31 ± 0.09 (pre) to 0.34 ± 0.10 post-sprinting (*p* = 0.02, d = 0.86) and 0.34 ± 0.09 (*p* = 0.04, d = 0.77) following recovery.

**Fig. 4:**
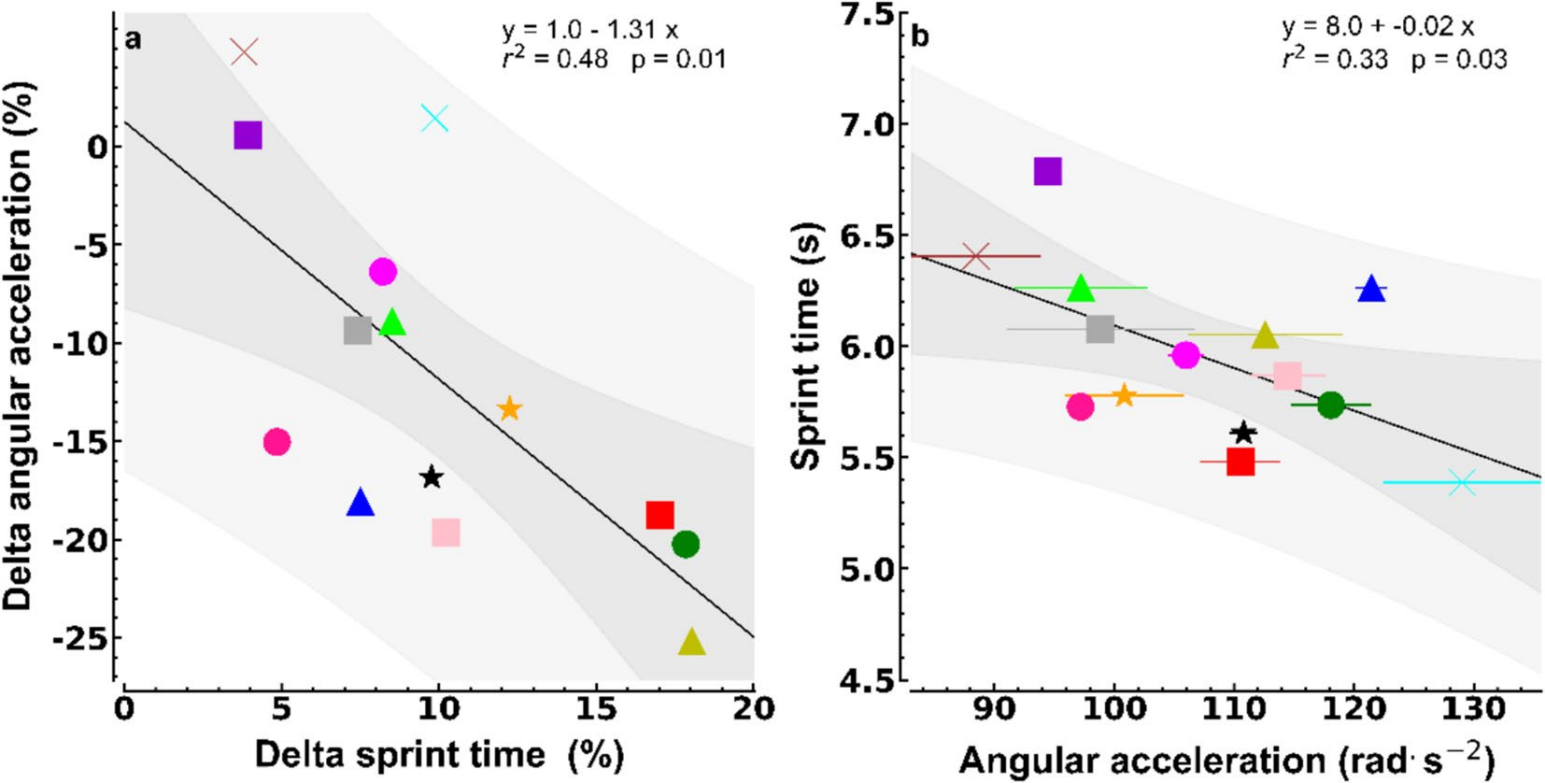
**a** Pre- to post-sprint changes in peak knee angular acceleration during FKFs as a function of the percentage changes in 40 m sprint time (x-axis). The Pre-sprint values are set to 100%. **b** Time of the first sprint as a function of pre-sprint peak knee angular acceleration during FKFs (mean values of three fastest attempts ± standard deviations) on the x-axis. Regression lines are shown with 95% confidence (dark grey) and prediction (light grey) intervals

As hypothesized, there was a significant relation between the fatigue-induced decreases in sprint performance (increased sprint times between sprint 1 and 14) and the decreases in PAA during the FKFs (Fig. 4a, *p* = 0.01, *r*^2^ = 0.48, excluding the female player: *p* = 0.02, *r*^2^ = 0.41).

There also was a significant linear relation (Fig. 4b, *p* = 0.03, *r*^2^ = 0.33, excluding the female player: *p* = 0.08, *r*^2^ = 0.26) between sprint and FKF performance in the unfatigued state.

## Discussion

The current findings are in line with our hypothesis. The results corroborate FKFs in a prone position being easy to perform and to standardize. Moreover, the decrease in sprint times during repetitive sprints varied among participants and was related to the decline in peak knee angular knee acceleration (Fig. 4a, *r*^2^ = 0.48).

The low CVs (~ 3%) of PAA, despite participants being unfamiliar with the FKF test and only practicing during the warm-up, is a very promising finding. Moreover, only 3 out of 210 (= 14 × 5x3) attempts were invalidated and this was for lifting the upper leg from the ground. Good within-session reproducibility of any test aiming to quantify changes during a training session or match is important because this enables the detection of relatively small changes in the variable of interest. The FKFs proved sensitive enough to detect a decrease of 12.0 ± 9.1% in PPA.

The test seems an asset in quantifying on-field knee flexor muscle fatigue. Similarly, vertical jumps have been proposed to assess on-field muscle fatigue rapidly after exercise (Jimenez-Reyes et al. 2019). However, jumps involve the coordinated contractions of several muscle groups, with knee extensors, gluteal, hamstring, and calf muscles all contributing to jump performance (Bobbert and van Ingen Schenau 1988). The FKF test specifically targets the knee flexors. We tried to limit calf muscle contribution to the knee flexions by starting with the foot in plantar flexion, but clearly, other muscles are involved in stabilization. Deformations of the vastus lateralis muscle were visible in the video recordings at the start of the knee flexions. The gluteal muscles may also be involved in movement stabilization. The extent of these co-contractions needs further investigation, but we are confident that the hamstring muscle group is the main determinant for FKF performance.

Hamstring injuries have been linked to fatigue and occur mostly acutely during running (Della Villa et al. 2024). Therefore, monitoring the state of knee flexor fatigue and subsequently adapting the training load or extending the recovery periods within and between sessions might reduce injury risk. It is impossible to assess the metabolic or contractile status of the knee flexors *during* sprinting, and following exercise recovery usually is rapid (Bogdanis et al. 1995; de Ruiter et al. 2000; Jones 2010; Bishop et al. 2011). Note that although the angle at which PAA was reached did not return (*p* < 0.05) to its pre-sprint value, a change of about 1.7 degrees probably is not meaningful.

The strength of the presented FKF test is, that in contrast to dynamometer testing, the fatigue assessment can be done within a handful of seconds after vigorous exercise on the field. In the present study, for reasons of standardization, we used a conservative 20 s after crossing the finish line of the last sprint. We assumed that recovery at this moment was still limited. Angular acceleration largely recovered after 5-6 min of rest as did sprint time. With the present set-up, we can only speculate that the main reasons for the reductions in PAA and sprint performance probably were of peripheral origin and were caused by intramuscular metabolic changes like a decrease in phosphocreatine, and an increase in inorganic phosphate, an increased proton concentration (Sundberg and Fitts 2019), and potentially interstitial potassium concentration (Lindinger and Cairns 2021). In addition to the direct interference of these metabolic by-products with cross-bridge function and sarcoplasmic calcium ion release (Sundberg and Fitts 2019), these changes feedback on the central nervous system via group III and IV afferents, making it plausible that decreased neural activation also contributed to the reduction of angular acceleration (Broxterman et al. 2018). Full recovery after a vigorous football match may take (more than) 72 h (Krustrup et al. 2011; Carmona et al. 2024). Whether FKF performance is sensitive to longer-lasting aspects of fatigue remains to be established. This would necessitate measuring across days and good reliability of the FKFs between different days should, therefore, be established.

The percentage decrease in peak knee angular acceleration during FKFs after the fatiguing sprints accounted for 48% of the variation in the increase in sprint time. We think this is quite a high percentage because the sprinting technique varied considerably among our participants and their pacing strategies probably also affected the changes in sprint time. In well-trained and very motivated sprinters sprint time would increase about linearly with sprint number and the final sprint would be the slowest (Jimenez-Reyes et al. 2019). However, our participants represent the average Dutch amateur football player, and notably different patterns were seen as illustrated by participants 7 and 14 in Fig. 2. In all but participant number 6, the last sprint was faster than the penultimate sprint of the series. This is often seen (van den Tillaar 2018) and relates to the end-spurt phenomenon (Foster et al. 2023). In the present study, the ‘end-spurt’ varied considerably with the last sprint being about 0.06 s faster in participants 1,3, and 10 while others (4,7,9,11,14) were more than 0.41 s faster than their penultimate sprint. Therefore, although all participants indicated that they felt fatigued post-sprinting (RPE values ≥ 8), it is likely that the performance decreases were also affected by motivational factors. Speculatively, FKF performance is less affected by motivation and pacing since short-lasting isolated kicks are experienced as far less demanding compared to a maximal 40 m sprint. Taking the above differences in sprint execution into account, the relation between the increases in sprint time and the decrease in PAA during the FKFs (Fig. 4a) seems quite convincing. It remains to be established whether the present knee flexion test can quantify different levels of knee flexor fatigue across days within individual players.

We did not anticipate finding a significant relation between sprinting, involving coordinated muscle contractions of many muscle groups, and PAA of the unfatigued knee flexors in isolation (*r*^2^ = 0.33). However, running speed is undoubtedly related to fast (lower) leg movements as good sprinters accommodate step frequencies of about 4.5 Hz (Debaere et al. 2013) necessitating very high knee angular speeds (Kivi et al. 2002). Thus, the presented relation between isolated maximal knee flexion acceleration and sprint speed may not be coincidental after all, however, it needs confirmation in larger studies preferably with sprint-trained athletes of different performance levels. In future studies, measuring EMG is recommended, since the capacity for maximal fast neuromuscular activation is an important factor in fast force development, and high PAAs expectantly are preceded by a high neural drive that would lead to high bursts of EMG activity at the onset of these explosive muscle contractions (de Ruiter et al. 2006; Tillin et al. 2010; Del Vecchio et al. 2019).

In conclusion, the proposed fast knee flexion test is reliable and can easily be executed to evaluate acute knee flexor muscle fatigue on the field. The presented relations between (changes in) sprint performance and peak knee angular accelerations during isolated fast knee flexions are interesting but need confirmation in other studies.

## Supplementary Information

Below is the link to the electronic supplementary material.

Supplementary file1 (XLSX 14 KB)

Supplementary file2 (XLSX 14 KB)

## Data Availability

In addition to the supplementary materials, the data that support the findings of this study are available from the corresponding author (C.J. de R.) upon reasonable request.

## Abbreviations

ANOVA: Analysis of variance
CV: Coefficient of variation
d: Cohen’s d effect size
f: Cohen’s f effect size
EMG: Electromyography
FKF(s): Fast knee flexion(s)
ICC: Intra class correlation coefficient
IMU: Inertial measurement unit
PAA: Peak angular acceleration
RPE: Rating of perceived exertion

## References

[CR1] Andersen LL, Andersen JL, Magnusson SP, Suetta C, Madsen JL, Christensen LR, Aagaard P (2005) Changes in the human muscle force-velocity relationship in response to resistance training and subsequent detraining. J Appl Physiol 99(1):87–94 15731398 10.1152/japplphysiol.00091.2005

[CR2] Arney BE, Glover R, Fusco A, Cortis C, de Koning JJ, van Erp T, Jaime S, Mikat RP, Porcari JP, Foster C (2019) Comparison of RPE (rating of perceived exertion) scales for session RPE. Int J Sports Physiol Perform 14(7):994–996. 10.1123/ijspp.2018-0637 30569764 10.1123/ijspp.2018-0637

[CR3] Askling CM, Tengvar M, Saartok T, Thorstensson A (2007) Acute first-time hamstring strains during high-speed running: a longitudinal study including clinical and magnetic resonance imaging findings. Am J Sports Med 35(2):197–206. 10.1177/0363546506294679 17170160 10.1177/0363546506294679

[CR4] Bishop D, Girard O, Mendez-Villanueva A (2011) Repeated-sprint ability - part II: recommendations for training. Sports Med 41(9):741–756. 10.2165/11590560-000000000-00000 21846163 10.2165/11590560-000000000-00000

[CR5] Bobbert MF, van Ingen Schenau GJ (1988) Coordination in vertical jumping. J Biomech 21(3):249–262 3379084 10.1016/0021-9290(88)90175-3

[CR6] Bogdanis GC, Nevill ME, Boobis LH, Lakomy HKA, Nevill AM (1995) Recovery of power output and muscle metabolites following 30 s of maximal sprint cycling in man. J Physiol 482:467–480 7714837 10.1113/jphysiol.1995.sp020533 PMC1157744

[CR7] Broxterman RM, Hureau TJ, Layec G, Morgan DE, Bledsoe AD, Jessop JE, Amann M, Richardson RS (2018) Influence of group III/IV muscle afferents on small muscle mass exercise performance: a bioenergetics perspective. J Physiol 596(12):2301–2314. 10.1113/JP275817 29644702 10.1113/JP275817 PMC6002240

[CR8] Carmona G, Moreno-Simonet L, Cosio PL, Astrella A, Fernandez D, Cadefau JA, Rodas G, Jou C, Milisenda JC, Cano MD, Aranega R, Marotta M, Grau JM, Padulles JM (2024) Mendiguchia J (2024) Hamstrings on focus: Are 72 hours sufficient for recovery after a football (soccer) match? A multidisciplinary approach based on hamstring injury risk factors and histology. J Sports Sci. 10.1080/02640414.2024.2386209 10.1080/02640414.2024.2386209 39087576

[CR9] de Ruiter CJ, van Dieen JH (2019) Stride and step length obtained with inertial measurement units during maximal sprint acceleration. Sports (Basel). 10.3390/sports7090202 10.3390/sports7090202 PMC6784208 31480457

[CR10] de Ruiter CJ, Didden WJ, Jones DA, de Haan A (2000) The force-velocity relationship of human adductor pollicis muscle during stretch and the effects of fatigue. J Physiol 526(Pt 3):671–681 10922017 10.1111/j.1469-7793.2000.00671.x PMC2270043

[CR11] de Ruiter CJ, Van Leeuwen D, Heijblom A, Bobbert MF, de Haan A (2006) Fast unilateral isometric knee extension torque development and bilateral jump height. Med Sci Sports Exerc 38(10):1843–1852 17019308 10.1249/01.mss.0000227644.14102.50

[CR12] de Ruiter CJ, Vermeulen G, Toussaint HM, de Haan A (2007) Isometric knee-extensor torque development and jump height in volleyball players. Med Sci Sports Exerc 39(8):1336–1346 17762367 10.1097/mss.0b013e318063c719

[CR13] de Ruiter CJ, Wilmes E, Brouwers SAJ, Jagers EC (2022) Concurrent validity of an easy-to-use inertial measurement unit-system to evaluate sagittal plane segment kinematics during overground sprinting at different speeds. Sports Biomech. 10.1080/14763141.2022.2056076 10.1080/14763141.2022.2056076 35353032

[CR14] Debaere S, Jonkers I, Delecluse C (2013) The contribution of step characteristics to sprint running performance in high-level male and female athletes. J Strength Cond Res 27(1):116–124. 10.1519/JSC.0b013e31825183ef 22395270 10.1519/JSC.0b013e31825183ef

[CR15] Del Vecchio A, Negro F, Holobar A, Casolo A, Folland JP, Felici F, Farina D (2019) You are as fast as your motor neurons: speed of recruitment and maximal discharge of motor neurons determine the maximal rate of force development in humans. J Physiol. 10.1113/JP277396 10.1113/JP277396 PMC6487919 30768687

[CR16] Della Villa F, Massa B, Bortolami A, Nanni G, Olmo J, Buckthorpe M (2023) Injury mechanisms and situational patterns of severe lower limb muscle injuries in male professional football (soccer) players: a systematic video analysis study on 103 cases. Br J Sports Med 57(24):1550–1558. 10.1136/bjsports-2023-106850 37898508 10.1136/bjsports-2023-106850

[CR17] Della Villa F, Buckthorpe M, Pellegrini A, Ranzini A, Esposito F, Crescenzo C, Nanni G, Zago M (2024) A comparative video analysis of hamstring injuries mechanism and situational pattern in men’s and women’s football (soccer). Knee Surg Sports Traumatol Arthrosc. 10.1002/ksa.12313 10.1002/ksa.12313 38881374

[CR18] Ekstrand J, Hagglund M, Walden M (2011) Epidemiology of muscle injuries in professional football (soccer). Am J Sports Med 39(6):1226–1232. 10.1177/0363546510395879 21335353 10.1177/0363546510395879

[CR19] Ekstrand J, Bengtsson H, Walden M, Davison M, Khan KM, Hagglund M (2022) Hamstring injury rates have increased during recent seasons and now constitute 24% of all injuries in men’s professional football: the UEFA Elite Club Injury Study from 2001/02 to 2021/22. Br J Sports Med 57(5):292–298. 10.1136/bjsports-2021-105407 36588400 10.1136/bjsports-2021-105407 PMC9985757

[CR20] Faul F, Erdfelder E, Lang AG, Buchner A (2007) G*Power 3: a flexible statistical power analysis program for the social, behavioral, and biomedical sciences. Behav Res Methods 39(2):175–191. 10.3758/bf03193146 17695343 10.3758/bf03193146

[CR21] Folland JP, Buckthorpe MW, Hannah R (2014) Human capacity for explosive force production: neural and contractile determinants. Scand J Med Sci Sports 24(6):894–906. 10.1111/sms.12131 25754620 10.1111/sms.12131

[CR22] Foster C, de Koning JJ, Hettinga FJ, Barroso R, Boullosa D, Casado A, Cortis C, Fusco A, Gregorich H, Jaime S, Jones AM, Malterer KR, Pettitt R, Porcari JP, Pratt C, Reinschmidt P, Skiba P, Splinter A, St Clair Gibson A, St Mary J, Thiel C, Uithoven K, van Tunen J (2023) Competition between desired competitive result, tolerable homeostatic disturbance, and psychophysiological interpretation determines pacing strategy. Int J Sports Physiol Perform 18(4):335–346. 10.1123/ijspp.2022-0171 36848906 10.1123/ijspp.2022-0171

[CR23] Houston ME, Norman RW, Froese EA (1988) Mechanical measures during maximal velocity knee extension exercise and their relation to fibre composition of the human vastus lateralis muscle. Eur J Appl Physiol 58:1–7 10.1007/BF00636595 3203652

[CR24] Jimenez-Reyes P, Pareja-Blanco F, Cuadrado-Penafiel V, Ortega-Becerra M, Parraga J, Gonzalez-Badillo JJ (2019) Jump height loss as an indicator of fatigue during sprint training. J Sports Sci 37(9):1029–1037. 10.1080/02640414.2018.1539445 30380362 10.1080/02640414.2018.1539445

[CR25] Jones DA (2010) Changes in the force-velocity relationship of fatigued muscle: implications for power production and possible causes. J Physiol 588(Pt 16):2977–2986. 10.1113/jphysiol.2010.190934 20547674 10.1113/jphysiol.2010.190934 PMC2956939

[CR26] Karatzaferi C, de Haan A, Ferguson RA, van Mechelen W, Sargeant AJ (2001) Phosphocreatine and ATP content in human single muscle fibres before and after maximum dynamic exercise. Pflügers Arch Eur J Physiol 442(3):467–474 11484780 10.1007/s004240100552

[CR27] Kivi DM, Maraj BK, Gervais P (2002) A kinematic analysis of high-speed treadmill sprinting over a range of velocities. Med Sci Sports Exerc 34(4):662–666. 10.1097/00005768-200204000-00016 11932576 10.1097/00005768-200204000-00016

[CR28] Koo TK, Li MY (2016) A guideline of selecting and reporting intraclass correlation coefficients for reliability research. J Chiropr Med 15(2):155–163. 10.1016/j.jcm.2016.02.012 27330520 10.1016/j.jcm.2016.02.012 PMC4913118

[CR29] Krustrup P, Ortenblad N, Nielsen J, Nybo L, Gunnarsson TP, Iaia FM, Madsen K, Stephens F, Greenhaff P, Bangsbo J (2011) Maximal voluntary contraction force, SR function and glycogen resynthesis during the first 72 h after a high-level competitive soccer game. Eur J Appl Physiol 111(12):2987–2995. 10.1007/s00421-011-1919-y 21448723 10.1007/s00421-011-1919-y

[CR30] Lindinger MI, Cairns SP (2021) Regulation of muscle potassium: exercise performance, fatigue and health implications. Eur J Appl Physiol 121(3):721–748. 10.1007/s00421-020-04546-8 33392745 10.1007/s00421-020-04546-8

[CR31] Madgwick SOH, Harrison AJL, Vaidyanathan R Estimation of IMU and MARG orientation using a gradient descent algorithm. In: 2011 IEEE International Conference on Rehabilitation Robotics, Zurich, Switzerland, 29 June-1 July 2011 2011. pp 1–7. 10.1109/ICORR.2011.5975346 10.1109/ICORR.2011.5975346 22275550

[CR32] Marshall PW, Lovell R, Jeppesen GK, Andersen K, Siegler JC (2014) Hamstring muscle fatigue and central motor output during a simulated soccer match. PLoS ONE 9(7):e102753. 10.1371/journal.pone.0102753 25047547 10.1371/journal.pone.0102753 PMC4105441

[CR33] Rahnama N, Reilly T, Lees A, Graham-Smith P (2003) Muscle fatigue induced by exercise simulating the work rate of competitive soccer. J Sports Sci 21(11):933–942. 10.1080/0264041031000140428 14626373 10.1080/0264041031000140428

[CR34] Sundberg CW, Fitts RH (2019) Bioenergetic basis of skeletal muscle fatigue. Curr Opin Physiol 10:118–127. 10.1016/j.cophys.2019.05.004 31342000 10.1016/j.cophys.2019.05.004 PMC6656370

[CR35] Tillin NA, Jimenez-Reyes P, Pain MT, Folland JP (2010) Neuromuscular performance of explosive power athletes versus untrained individuals. Med Sci Sports Exerc 42(4):781–790. 10.1249/MSS.0b013e3181be9c7e 19952835 10.1249/MSS.0b013e3181be9c7e

[CR36] van den Tillaar R (2018) Comparison of step-by-step kinematics in repeated 30-m sprints in female soccer players. J Strength Cond Res 32(7):1923–1928. 10.1519/JSC.0000000000002429 29337832 10.1519/JSC.0000000000002429

[CR37] Vollestad NK (1997) Measurement of human muscle fatigue. J Neurosci Methods 74(2):219–227 9219890 10.1016/s0165-0270(97)02251-6

[CR38] Wilmes E, de Ruiter CJ, Bastiaansen BJC, van Zon JF, Vegter RJK, Brink MS, Goedhart EA, Lemmink K, Savelsbergh GJP (2020) Inertial sensor-based motion tracking in football with movement intensity quantification. Sensors (Basel). 10.3390/s20092527 10.3390/s20092527 PMC7248913 32365622

[CR39] Wilmes E, de Ruiter CJ, Bastiaansen BJC, Goedhart EA, Brink MS (2021) Associations between Hamstring Fatigue and Sprint Kinematics during a Simulated Football (Soccer) Match. Med Sci Sports Exerc 53(12):2586–2595. 10.1249/MSS.0000000000002753 34265817 10.1249/MSS.0000000000002753 PMC8594518

[CR40] Woods C, Hawkins RD, Maltby S, Hulse M, Thomas A, Hodson A (2004) The Football Association Medical Research Programme: an audit of injuries in professional football–analysis of hamstring injuries. Br J Sports Med 38(1):36–41. 10.1136/bjsm.2002.002352 14751943 10.1136/bjsm.2002.002352 PMC1724733

